# Endoscopic palliation of malignant dysphagia: a challenging task in inoperable oesophageal cancer

**DOI:** 10.1186/1477-7819-4-38

**Published:** 2006-07-04

**Authors:** IE Katsoulis, A Karoon, S Mylvaganam, JI Livingstone

**Affiliations:** 1 Upper Gastrointestinal Surgery Unit, Watford General Hospital and Mount Vernon Centre for Cancer, Northwood, London, UK

## Abstract

**Background:**

The main goal when managing patients with inoperable oesophageal cancer is to restore and maintain their oral nutrition. The aim of the present study was to assess the value of endoscopic palliation of dysphagia in patients with oesophageal cancer, who either due to advanced stage of the disease or co-morbidity are not suitable for surgery.

**Patients and methods:**

All the endoscopic palliative procedures performed over a 5-year period in our unit were retrospectively reviewed. Dilatation and insertion of self-expandable metal stents (SEMS) were mainly used for tight circumferential strictures whilst ablation with Nd-YAG laser was used for exophytic lesions. All procedures were performed under sedation.

**Results:**

Overall 249 palliative procedures were performed in 59 men and 40 women, with a median age of 73 years (range 35 – 93). The median number of sessions per patient was 2 (range 1 – 13 sessions). Palliation involved laser ablation alone in 24%, stent insertion alone in 22% and dilatation alone in 13% of the patients. In 41% of the patients, a combination of the above palliative techniques was applied. A total of 45 SEMS were inserted. One third of the patients did not receive any other palliative treatment, whilst the rest received chemotherapy, radiotherapy or chemoradiotherapy. Swallowing was maintained in all patients up to death. Four oesophageal perforations were encountered; two were fatal whilst the other two were successfully treated with covered stent insertion and conservative treatment. The median survival from diagnosis was 10.5 months (range 0.5–83 months) and the median survival from 1^st ^palliation was 5 months (range 0.5–68.5 months).

**Conclusion:**

Endoscopic interventions are effective and relatively safe palliative modalities for patients with oesophageal cancer. It is possible to adequately palliate almost all cases of malignant dysphagia. This is achieved by expertise in combination treatment.

## Background

Oesophageal cancer is a relatively rare disease. The average age standardised incidence rate in the EU for the year 2004, was 9.5 per 100,000 of male population and 8.7 per 100,000 of female population [1]. Patients with oesophageal cancer and cancer of the gastro-oesophageal junction (GOJ) commonly present with advanced disease mainly because the oesophagus is quite distentable and patients may not experience dysphagia until almost half of the luminal diameter is compromised. At least 60% of patients with oesophageal cancer will be unsuitable for surgical resection either due to the stage of their disease or due to co-morbidity [2,3]. A large proportion of patients treated surgically, on the other hand, despite curative intention will turn out to have undergone a palliative resection [4]. The mean survival in these patients is only 4 – 6 months and the aim of their management is to improve the quality of life with palliative treatment.

Dysphagia is the most devastating symptom and is classified into four grades of severity [2]. Aspiration is another frequent symptom requiring palliative therapy. This is either aspiration of saliva as a result of complete dysphagia or food aspiration due to a tracheo-oesophageal fistula.

Palliative bypass surgery, which was widely performed until the late 80's, has been discounted because of its high mortality rates in the range of 20–35% [5,6]. Nowadays, main palliative modalities include radiotherapy, endoluminal ablative therapies and insertion of endoprostheses [3,5,7-12]. This study looked specifically at the modalities of laser ablation and self-expanding metal stents (SEMS). The aim was to assess the value and effectiveness of these procedures in palliation of malignant dysphagia.

## Patients and methods

We retrospectively reviewed all the endoscopic palliative procedures performed in our Unit between January 1999 and December 2003. Overall 249 palliative procedures were performed in 99 patients, 59 men and 40 women, with a median age of 73 years (range 35–93 years). The characteristics of the tumours, the degree of dysphagia, the type of palliation, the complications and the overall survival were recorded.

In patients with short strictures, a mild dilatation of the malignant stricture to 12–14 mm with flexible rubber Savary-Guillard dilators was performed as the primary palliative treatment. The insertion of a self-expandable metal stent (SEMS) was mainly used for long tight circumferential strictures and in strictures that did not respond to other type of palliative treatment. In very frail patients a SEMS was often applied as the single palliative modality. Other indications for SEMS insertion were cases of oesophageal perforation. The type of SEMS used in this series was the *Ultraflex (Boston Scientific, MA, USA)*. This stent is constructed of a single strand of nitilol wire that exerts a constant, gentle radial pressure while minimising traumatic tissue compression and comes in a range of lengths (7–15 cm) and diameters (17–22 mm). These stents are available with both proximal-release and distal-release deployment systems. In our practice, proximal-release stents were used for strictures of the middle and upper oesophagus whilst distal-release stents were used for strictures of the gastro-oesophageal junction. All the stents were inserted under endoscopic control.

Endoluminal ablation with neodymium-yttrium-aluminum-garnet (Nd-YAG) laser was mainly used for exophytic lesions or short strictures (*Sharlan 2100, Litechnica Ltd, Middlesex, UK*). Laser output was set at 60 – 90 W and 1- to 2-second pulses were delivered to the tumour. Laser ablation was performed with the retrograde approach i.e. from distally to proximally. The total amount of energy varied according to the local conditions and the patient's tolerance.

More than one session and combination of techniques was often necessary. All the procedures were performed under sedation. The sedation protocol included intravenous administration of diazepam emulsion preparation 5 mg/ml (*Diazemuls*). Oxygen was also administered and monitoring during the procedure comprised pulse oxymetry. The vast majority of cases were performed as day cases and the patient after recovery was sent home with appropriate instructions.

## Results

**Figure 1:**
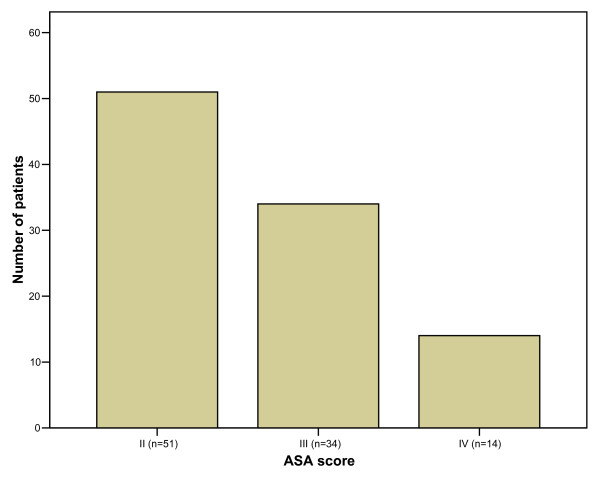
ASA score of patients.

**Figure 2:**
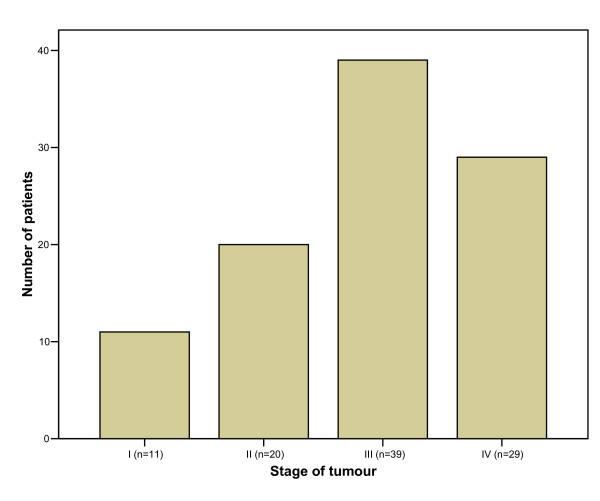
Stage of oesophageal carcinoma.

There were 3 upper third, 22 middle third, 41 lower third oesophageal tumours and 33 GOJ tumours. Sixty-three of these tumours were adenocarcinomas, 35 were squamous cell carcinomas and a GOJ tumour was a lymphoma. According to Müller's classification, 36 patients had grade 1 dysphagia, 29 had grade 2, 22 had grade 3 and 12 patients had grade 4 dysphagia. The patients of the present study were unsuitable for surgery either due to comorbidities or due to the stage of their disease. Figure 1 shows the patients' ASA classification and figure 2 shows the stage of their disease.

**Figure 3:**
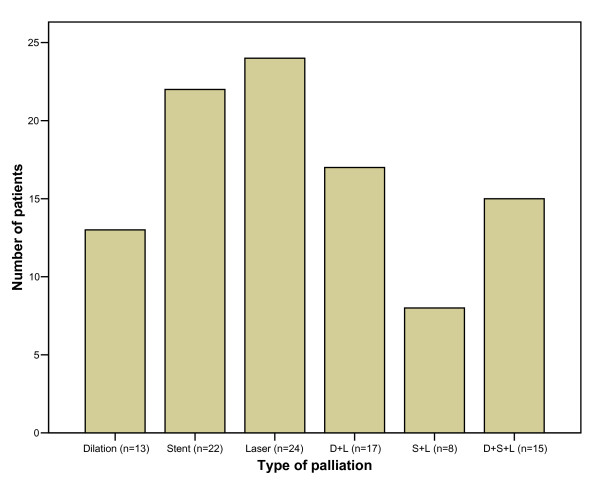
Endoscopic modalities used to palliate dysphagia.

**Figure 4:**
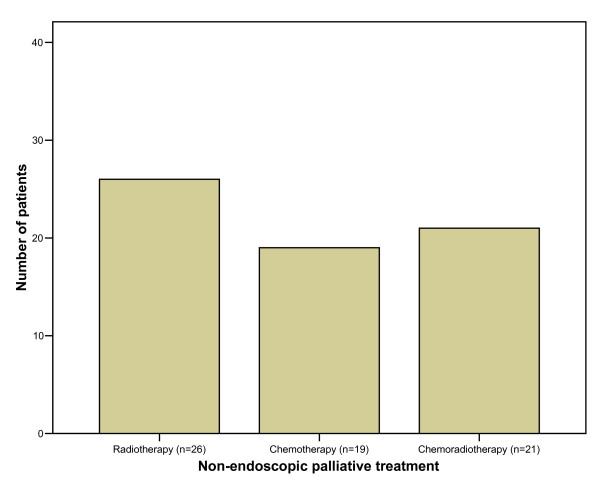
Other type of non-endoscopic palliative therapy was also used in 2/3 of patients.

Overall 249 palliative procedures were performed. The median number of palliative endoscopic sessions per patient was 2 (range 1 – 13 sessions). In 13 patients (13%) one or more dilatations were used as the single palliative modality. Palliation involved laser ablation alone in 24% and stent insertion alone in 22% of the patients. In 41% of the patients, a combination of the above palliative techniques was applied (Fig. 3). A total of 45 SEMS were inserted with a median length of 12 cm (range 9 – 15 cm). Treatment involved laser ablation in 61 patients with a median energy of 2442 Joules per session (range 60 – 10991 Joules). Two thirds of the patients were also given non-endoscopic palliative treatment for controlling the tumour in the form of chemotherapy, radiotherapy or chemoradiotherapy (Fig. 4). The radiotherapy in all patients was external beam irradiation.

In 24 patients that laser ablation was used as monotherapy, the time interval between sessions was usually 4–6 weeks depending on the response and the patient's status. In other 16 patients, however, where despite repetition of Nd-YAG laser ablation treatment dysphagia persisted, a SEMS was eventually placed. The median number of laser treatment sessions before insertion of SEMS in this group of patients was 2 (range 1 – 5 sessions).

Swallowing was maintained in all patients up to death. Four oesophageal perforations were encountered; two were fatal whilst the other two were successfully treated with covered stent insertion and conservative treatment. The first of sthe fatal perforations followed a dilatation of a tight GOJ tumour to 14 mm. The second fatal perforation followed laser ablation of a middle oesophageal tumour. Both patients developed severe mediastinitis and deteriorated rapidly. Due to their terminal disease and frailty further interventions were deemed inappropriate and they were kept comfortable until their death.

Another oesophageal perforation was caused during dilatation of a very tight GOJ tumour to 12 mm. On reinspection a posterior oesophageal rupture was seen and a covered SEMS was immediately placed. Lastly, there was a suspected small perforation during a SEMS placement for a lower third oesophageal tumour. Both these patients were treated conservatively and commenced oral intake a few days later after a gastrograffin swallow test.

A patient with severe ischaemic heart disease died from myocardial infarction on day 6 following the insertion of a SEMS. Two further patients died of their disease within a month following a session of endoscopic intervention. These deaths, however, should not be considerd related to the procedure that was performed.

In two patients the session of laser ablation treatment had to be terminated due to uncontrolled coughing fit and was postponed to a later date.

Problems related to the placement of SEMS were food bolus impaction and occlusion of the stent in 5 patients, excessive tumour ingrowth in 6 patients and encroachment of stent over GOJ in one patient. The blockage was treated according to the local conditions with either just balloon dilatation or laser treatment. In two of these patients local recurrence of the tumour occluded the lumen almost completely and a new stent had to be placed. Lastly, there was a case of persisting dysphagia following the placement of a stent, despite free contrast flow on barium meal. It was assumed that this type of functional dysphagia was a result of neuromuscular dysfunction secondary to tumour infiltration.

The median survival from diagnosis was 10.5 months (range 0.5 – 83 months) and the median survival from 1^st ^palliation was 5 months (range 0.5 – 68.5 months).

The median time from diagnosis to first palliation was 3 months (range 0 – 47.5 months).

## Discussion

The main goal when managing patients with inoperable oesophageal and junctional cancer is to restore and maintain their oral nutrition. Furthermore, aims of palliation should also be minimizing the hospital stay, relief of pain, elimination of reflux and regurgitation, and the prevention of aspiration [7]. A variety of options to palliate dysphagia have been developed and evolved in recent years [3,5,7-12]. The choice for palliation for a particular patient will depend on tumour size and location, the experience of the operator and the resources available at the health care institution.

Since 1999 in our practice at Mount Vernon Centre for Cancer, palliative modalities for malignant dysphagia include insertion of SEMS and endoluminal ablation with Nd-YAG laser.

SEMS were introduced in the early 1990s and have nowadays superseded conventional endoluminal oesophageal prostheses despite their high cost which is at the range of £700–800. Their major advantages are ease of placement, immediate relief and low perforation risk as compared to conventional prostheses. Potential problems related to SEMS may be migration of the stent, food bolus impaction, gastro-oesophageal reflux and tumour ingrowth into the stent mesh or around the ends of the stent. In our series food occluded the stent in 5 patients and tumour ingrowth occurred in 6 patients. Gastro-oesophageal reflux was an issue in a few cases of GOJ stents; however all patients responded well to treatment with proton pump inhibitors (PPIs). No stent migration or other delayed complication occurred.

The introduction of endoluminal tumour ablation with Nd-YAG laser for exophytic oesophageal or GOJ malignancies was at one time a major advance in therapeutic endoscopy as it dramatically reduced the number of conventional oesophageal prostheses placed. Endoluminal tumour ablation with Nd-YAG laser provides immediate dysphagia relief but has limited durability (weeks) if not followed by adjuvant therapy [8]. We usually repeat ablation every 4–6 weeks. From technical point of view, we use the retrograde approach and find it effective and safe. Mitty et al [9] reported the treatment of 62 patients with Nd-YAG laser ablation and concluded that the retrograde approach allowed fewer treatment sessions, the treatment of longer lesions, greater improvement in dysphagia and reduced the risk of aspiration.

This was a retrospective study with all its inherent shortcomings. Our results showed that the use of one or a combination of these interventions enabled swallowing to be maintained in all patients until death. We could not, however, quantify in retrospect the improvement of dysphagia and to assess the impact on the patients' quality of life.

The choice of the most appropriate treatment at every session should be tailored to the type of stricture and the patient's condition. In this series, the insertion of SEMS was mainly used for long tight circumferential strictures and in strictures that did not respond to other type of palliative treatment. In very frail patients SEMS placement was often applied as the single palliative modality. Endoluminal ablation with Nd-YAG laser was mainly used for exophytic lesions or short strictures. In 24 patients laser ablation was used as monotherapy effectively. In other 16 patients, however, where despite repetition of laser treatment dysphagia persisted, a SEMS was eventually placed. The number of laser ablation sessions and the amount of energy required varied and the eventual decision of a stent placement relied both upon the response and the patient's status.

Another issue is dealing with problems that may ensue after placement of a stent. The operator has to be able to tackle proficiently a variety of conditions including complications using a combination of techniques. In the present series occlusion of the stent either due to food impaction or due to tumour local overgrowth was treated according to the local conditions with balloon dilatation or laser ablation and in two cases with insertion of an extra stent.

This study has also shown that endoscopic interventions can be performed as day case procedures under sedation, providing that hospital backup is readily available.

Overall survival in this study did not significantly differ from figures in the literature whereas complication rates were much lower than previously reported in other series [9,10,12]. Palliation of malignant strictures involves specialized procedures and therefore expertise developed in regional centres, which could minimize complication rates [7].

## Conclusion

It is possible to adequately palliate almost all cases of malignant dysphagia. This is achieved by expertise in combination treatment. Palliation of malignant dysphagia by the use of laser ablation and SEMS insertion is effective and relatively safe. The management of patients with oesophagogastric cancer, who are not suitable for surgery, requires a multidisciplinary approach in specialised cancer centres.

## Competing interests

The author(s) declare that they have no competing interests.

## Authors' contributions

IEK conceived the study, participated in the design of the study, performed the analysis and drafted the manuscript. AK and SM participated in the design of the study and collected the data. JIL, director of the unit, helped to draft the manuscript. All authors read and approved the final manuscript.
